# Effect of Spironolactone in Resistant Arterial Hypertension: A Randomized, Double-Blind, Placebo-Controlled Trial (ASPIRANT-EXT)

**DOI:** 10.1097/MD.0000000000000162

**Published:** 2014-12-12

**Authors:** Jan Václavík, Richard Sedlák, Jiří Jarkovský, Eva Kociánová, Miloš Táborský

**Affiliations:** From the Department of Internal Medicine I—Cardiology, Faculty of Medicine and Dentistry, University Hospital Olomouc and Palacký University, Olomouc (JV, EK, MT); Department of Internal Medicine, Prostějov Hospital, Mathonova, Prostějov (RS); and Institute of Biostatistics and Analyses at the Faculty of Medicine and the Faculty of Science of the Masaryk University, Kamenice, Brno, Czech Republic (JJ).

## Abstract

This study was designed to assess the effect of the addition of low-dose spironolactone on blood pressure (BP) in patients with resistant arterial hypertension.

Patients with office systolic blood pressure (SBP) >140 mm Hg or diastolic blood pressure (DBP) >90 mm Hg despite treatment with at least 3 antihypertensive drugs, including a diuretic, were enrolled in this double-blind, placebo-controlled, multicentre trial. One hundred sixty-one patients in outpatient internal medicine departments of 6 hospitals in the Czech Republic were randomly assigned to receive 25 mg of spironolactone (N = 81) or a placebo (N = 80) once daily as an add-on to their antihypertensive medication, using simple randomization. This study was registered with ClinicalTrials.gov, number NCT00524615.

Analyses were done with 150 patients who finished the follow-up (74 in the spironolactone and 76 in the placebo group). At 8 weeks, BP values were decreased more by spironolactone, with differences in mean fall of SBP of −9.8, −13.0, −10.5, and −9.9 mm Hg (*P* < 0.001 for all) in daytime, nighttime, and 24-hour ambulatory BP monitoring and in the office. The respective DBP differences were −3.2, −6.4, −3.5, and −3.0 mm Hg (*P* = 0.013, *P* < 0.001, *P* = 0.005, and *P* = 0.003). Adverse events in both groups were comparable. The office SBP goal <140 mm Hg at 8 weeks was reached in 73% of patients using spironolactone and 41% using placebo (*P* = 0.001).

Spironolactone in patients with resistant arterial hypertension leads to a significant decrease of both SBP and DBP and markedly improves BP control.

## Abstract 2

Supplemental Digital Content is available in the text

## INTRODUCTION

Resistant hypertension is a common clinical problem faced by both primary care clinicians and specialists worldwide. It is defined as blood pressure (BP) that remains above goal in spite of the concurrent use of 3 antihypertensive agents of different classes prescribed at optimal dosages; 1 of the 3 agents used should be a diuretic.^1^

The prevalence of resistant hypertension has varied from 9.5% to 12.8% in recent reports.^2–4^ If no secondary cause of hypertension is found, multidrug treatment regimens including 3, 4, or more antihypertensive drugs are usually necessary to lower BP and, thus, lower the risk of future cardiovascular events.

Spironolactone is a mineralocorticoid receptor antagonist. In low doses (median 25 mg daily), it has been shown to substantially lower BP in several uncontrolled trials in resistant arterial hypertension, leading to a significant reduction of systolic blood pressure (SBP) between 14 and 36 mm Hg and diastolic between 7 and 12.5 mm Hg.^5–11^ The BP lowering effect of spironolactone was shown to be markedly larger than that of other antihypertensive drugs,^7^ doxazosin^12^ or dual blockade of the renin-angiotensin-aldosterone system.^13^ However, various confounding factors could have significantly influenced the results of the previous trials with spironolactone and with the absence of a control group, the cause and effect relationship, as well as safety, could not be established.^10,14^

Because evidence from randomized trials was necessary to provide proof for the efficacy of spironolactone as an add-on treatment in resistant hypertension,^9,15^ we designed and performed a prospective randomized trial (Addition of Spironolactone in Patients with Resistant Arterial Hypertension [ASPIRANT]) to evaluate the effect of adding 25 mg spironolactone in patients with resistant arterial hypertension.^16,17^ We decided to administer a low dose of spironolactone 25 mg/day in the trial, as the effect of this dose seemed to be substantial according to data from previous trials, and we wanted to avoid possible adverse effects.

After being stopped prematurely after the first interim analysis with 117 enrolled patients, the ASPIRANT trial showed a significant decrease of systolic, but not diastolic blood pressure (DBP) with spironolactone treatment.^17^ After several discussions with other experts, we decided to perform an extension of this trial (ASPIRANT-EXT) by continuing the enrollment with the same inclusion and exclusion criteria to include the total number of participants that had been planned at the beginning of the trial.

## METHODS

### Study Design and Population

ASPIRANT-EXT was an investigator-led, prospective, multicenter, randomized, double-blind, placebo-controlled, parallel-group trial. The design of the trial has been described previously.^16,17^ We enrolled patients >18 years with resistant arterial hypertension. Resistant hypertension was defined as office SBP >140 or DBP >90 mm Hg despite being treated with at least 3 antihypertensive drugs, including a diuretic. Patients with diabetes or chronic kidney disease (defined as serum creatinine >133 μmol/L or proteinuria >300 mg/day) were enrolled if the office BP was >130/80 mm Hg.

The study was done in accordance with the principles of the Helsinki declaration. The study protocol was approved by the ethical review committees at all 6 participating secondary or tertiary care centers and by the State Institute for Drug Control of the Czech Republic. Written informed consent was obtained from all patients before enrollment. This study was registered at clinicaltrials.gov as NCT00524615 and the EudraCT number of the trial was 2007-003558-27.

For safety reasons we excluded all patients with severe hypertension (SBP >180 or DBP >110 mm Hg) who needed an immediate adjustment of treatment, renal insufficiency with serum creatinine >180 μmol/L or glomerular filtration rate <40 mL/minute calculated by the Modification of Diet in Renal Disease formula,^18^ hyperkalemia >5.4 mmol/L, hyponatremia <130 mmol/L, porphyria, pregnant or lactating women, or women of fertile age not using effective contraception, patients with known prior hypersensitivity to the drug Verospiron (spironolactone; Richter Gedeon Ltd., Czech Republic) or currently using any aldosterone antagonist (spironolactone, eplerenone, or canreonate). Patients were not enrolled if a secondary cause of hypertension was found before randomization.

### Procedures

Patients were randomly assigned in a 1 : 1 ratio to receive either spironolactone at a dose of 25 mg once daily or a placebo once daily in the morning, as an add-on to their current antihypertensive therapy, using simple randomization without stratification. Patients received blinded study medication in phials marked with different colors in a random manner. The Olomouc University Hospital Pharmacy Department prepared the study medication containing spironolactone or placebo as identical looking gelatinous capsules, placed into glass containers similar in weight and appearance. This department also held the randomization codes, which were disclosed after the study. The individual who prepared the blinded color-marked containers of drugs was not otherwise connected to the study, and all the investigators and patients were blinded to patient assignment and medication during the whole study period. All the study investigators deemed the blinding to be adequate and sufficient throughout the study.

After randomization, visits were scheduled at 4 and 8 weeks. In patients with diabetes, patients >75 years and with serum creatinine >133 μmol/L, an additional safety visit was performed 2 weeks after randomization. During every visit, office BP was recorded by a calibrated mercury sphygmomanometer in seated patients with their arm supported. The value was recorded as the average of the second and third measurements with a minimum delay of 3 minutes between the measurements. At baseline and 8 weeks, 24-hour ambulatory blood pressure monitoring (ABPM) was performed with validated devices with BP measurements programmed every 20 to 30 minute.^14,19^ Average daytime BP was calculated from values measured between 09:00 and 21:00 hour, average nighttime BP from values measured between 01:00 and 06:00 hour, and average 24-hour BP was calculated from all the values recorded by ABPM.^20^

Serum sodium, potassium, chloride, urea and creatinine, body weight, and pulse were measured during every visit. Plasma renin activity (PRA), plasma aldosterone and aldosterone/renin ratio (ARR), albuminuria, and proteinuria in a 24-hour urine sample were measured at baseline and at 8 weeks. The blood samples for PRA and aldosterone were collected in the morning, after the patients had been seated for 5 to 15 minute, without discontinuation of the medications.^21^ Antihypertensive medications and all other medications were recorded at baseline and patients did not change doses or the number of their antihypertensive medications throughout the trial. At every visit, patients were asked about the occurrence of any adverse effects of the medication. Compliance of patients was assessed by the calculation of returned tablets.

According to study protocol, the administration of randomized medication was to be terminated at any time in case of symptomatic hypotension <100/60 mm Hg, increase of serum potassium >6.0 mmol/L, increase of serum creatinine >25% compared with baseline and exceeding the upper reference limit of 104 μmol/L, if the patient did not tolerate the study medication because of adverse effects or any other reason, or if the patient withdrew informed consent.

Our primary end-point was the comparison of the fall of daytime systolic and diastolic pressure on ABPM between the spironolactone and placebo groups after 8 weeks of treatment. The secondary end-points were the comparison of the fall of 24-hour and nighttime systolic and DBP and office BP between the spironolactone and placebo groups after 8 weeks of treatment. Further secondary end-points were to compare the changes of serum levels of sodium, potassium and creatinine, and body weight between treatment groups and to evaluate the response to spironolactone treatment based on the baseline potassium, PRA, aldosterone level and baseline ARR.

### Statistical Analysis

Standard descriptive statistics were applied in the analysis. Continuous variables were described using mean and standard deviation (SD) when the prerequisite of normality was fulfilled and the median and 5th and 95th percentile range in the case of nonnormal distribution. Categorical variables were described by the number of cases and the percentages of categories. The statistical significance of differences between study groups was analyzed by the Mann–Whitney *U* test for continuous variables and the Fisher exact test for categorical variables. Change in BP between the groups was calculated by the Mann–Whitney *U* test of the difference from baseline. Statistical analysis was computed using SPSS 18.0.2 (IBM Corporation, New York, NY).

Power calculations were based on an expected average difference of SBP fall between spironolactone and the placebo 10 mm Hg (SD 18.0 mm Hg) and DBP fall difference 5 mm Hg (SD 10.7 mm Hg).^9^ We needed a total of 102 patients to have 90% power for SBP and 146 patients for DBP at *P* < 0.05. We expected about 90% of the randomized patients to complete the trial and, therefore, planned to recruit 160 patients.^16^

### Role of the Funding Source

The study sponsors only provided financial support; they were not involved in the study design, had no role in the collection, analysis, or interpretation of the data and were not involved in decisions about its publication. The corresponding author had full access to all data and had final responsibility for the decision to submit the study for publication.

## RESULTS

**FIGURE 1:**
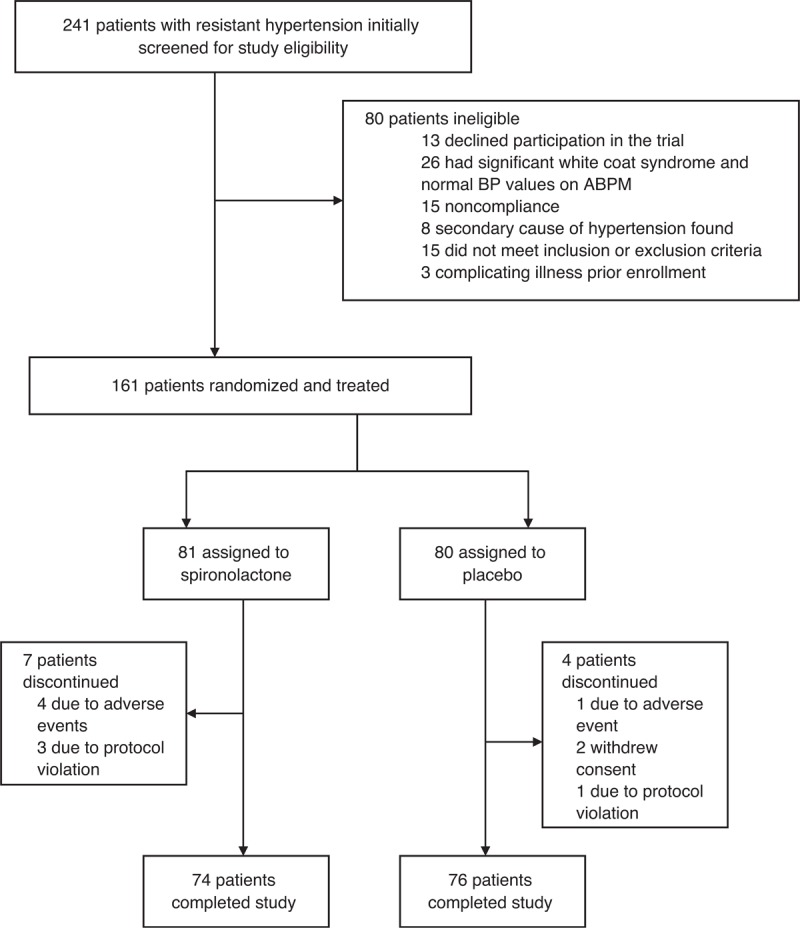
Trial profile. ABPM = ambulatory blood pressure monitoring, BP = blood pressure.

The trial profile is shown in Figure 1. Patients were recruited from September 25, 2007, through August 22, 2012, with follow-up over the 2 following months. Of the 241 screened patients, 161 (67%) were eligible for enrollment; 80 (33%) patients were not included for reasons specified in Figure 1. The enrollment was stopped after the planned number of patients was recruited.

Baseline characteristics were well matched between the treatment groups in baseline demographic characteristics, mean baseline BPs, baseline serum and urinary laboratory characteristics, and antihypertensive medication, with the exception of higher baseline glycemia and higher diabetes mellitus prevalence in the placebo group (Table 1).

**TABLE 1:**
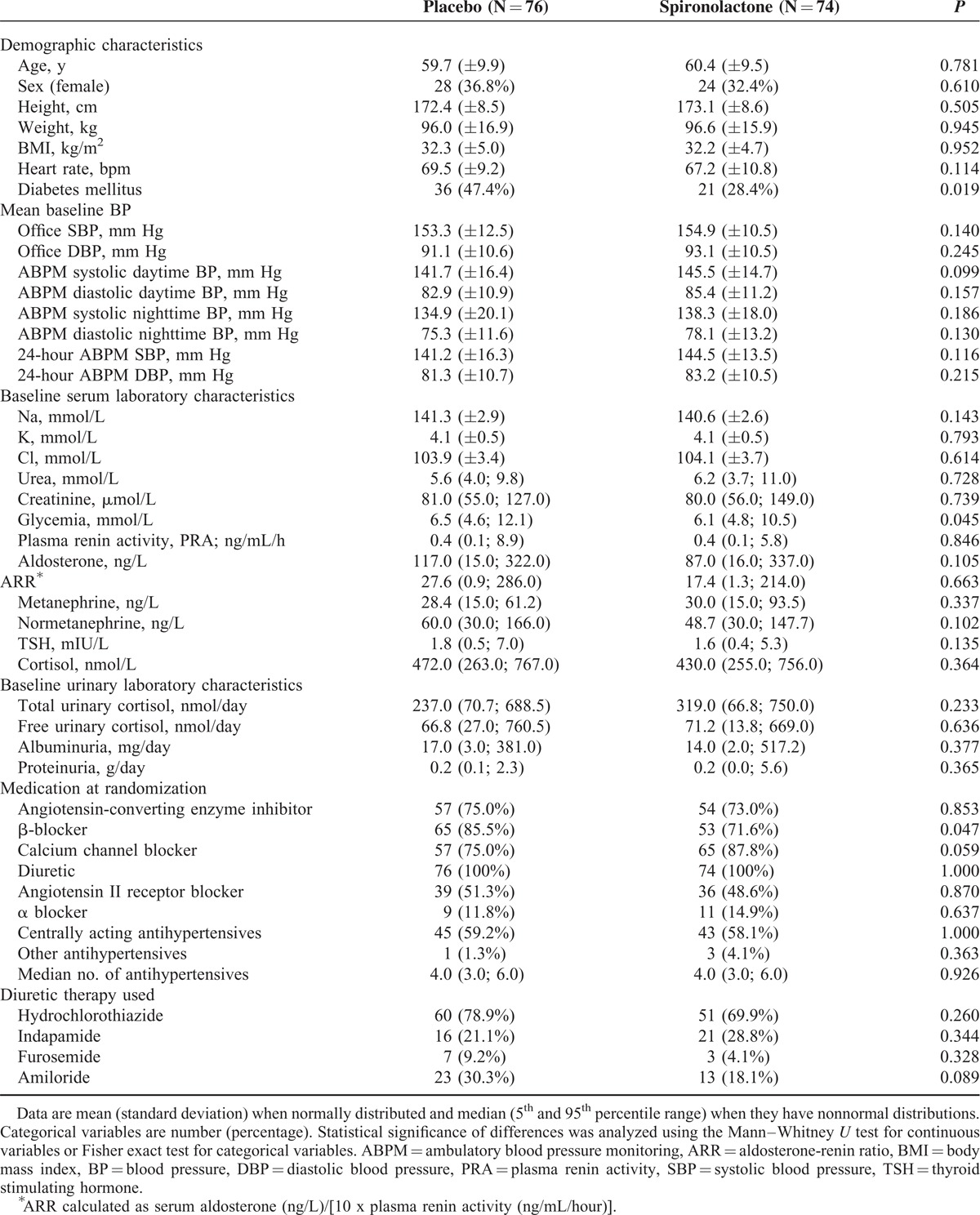
Patient Demographics and Baseline Characteristics

The mean age of patients was approximately 60 years, heart rate was 68 beats per minute and body mass index was 32.3 kg/m^2^. Mean office BP was 154/92 mm Hg, daytime ABPM BP was 143/84 mm Hg, 24-hour ABPM BP was 143/82 mm Hg. Isolated systolic hypertension (office SBP >140 mm Hg and DBP <90 mm Hg) was present in 35% of patients in the spironolactone group and 38% of patients in the placebo group. Patients were using a mean of 4.5 antihypertensive drugs (median 4.0) in both the spironolactone and placebo groups. Most patients used either hydrochlorothiazide or indapamide. A small number of patients used a combination of more diuretics, such as hydrochlorothiazide with amiloride, hydrochlorothiazide with furosemide, or indapamide with furosemide.

**FIGURE 2:**
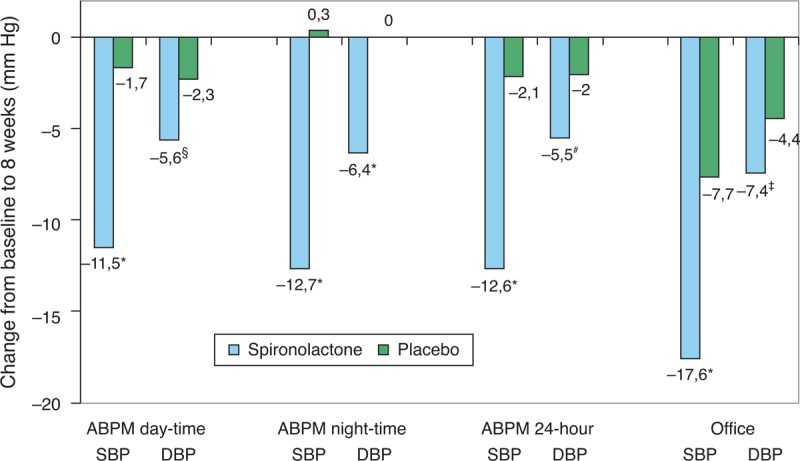
Paired changes of SBP and DBP from baseline to 8 weeks for spironolactone and placebo groups. ABPM = ambulatory blood pressure monitoring, DBP = diastolic blood pressure, SBP = systolic blood pressure. Statistical significance of the difference between spironolactone and placebo groups is marked with the following symbols: ^∗^*P* < 0.001, ^§^*P* = 0.013, ^#^*P* = 0.005, ^‡^*P* = 0.003.

The change of BP values after 8 weeks of treatment compared with baseline is shown in Table 2 and Figure 2. The difference between the fall of mean ABPM daytime SBP between the spironolactone and placebo groups was −9.8 mm Hg (95% CI −14.2; −5.4, *P* < 0.001) for systolic and −3.2 mm Hg (95% CI −5.9; −0.5, *P* = 0.013) for DBP. The APBM nighttime systolic, 24-hour ABPM systolic, and office SBP values were significantly decreased by spironolactone, as were the respective DBP values (Table 2). Spironolactone significantly reduced pulse pressure in all of the ABPM and office measurements (Table 2).

**TABLE 2:**
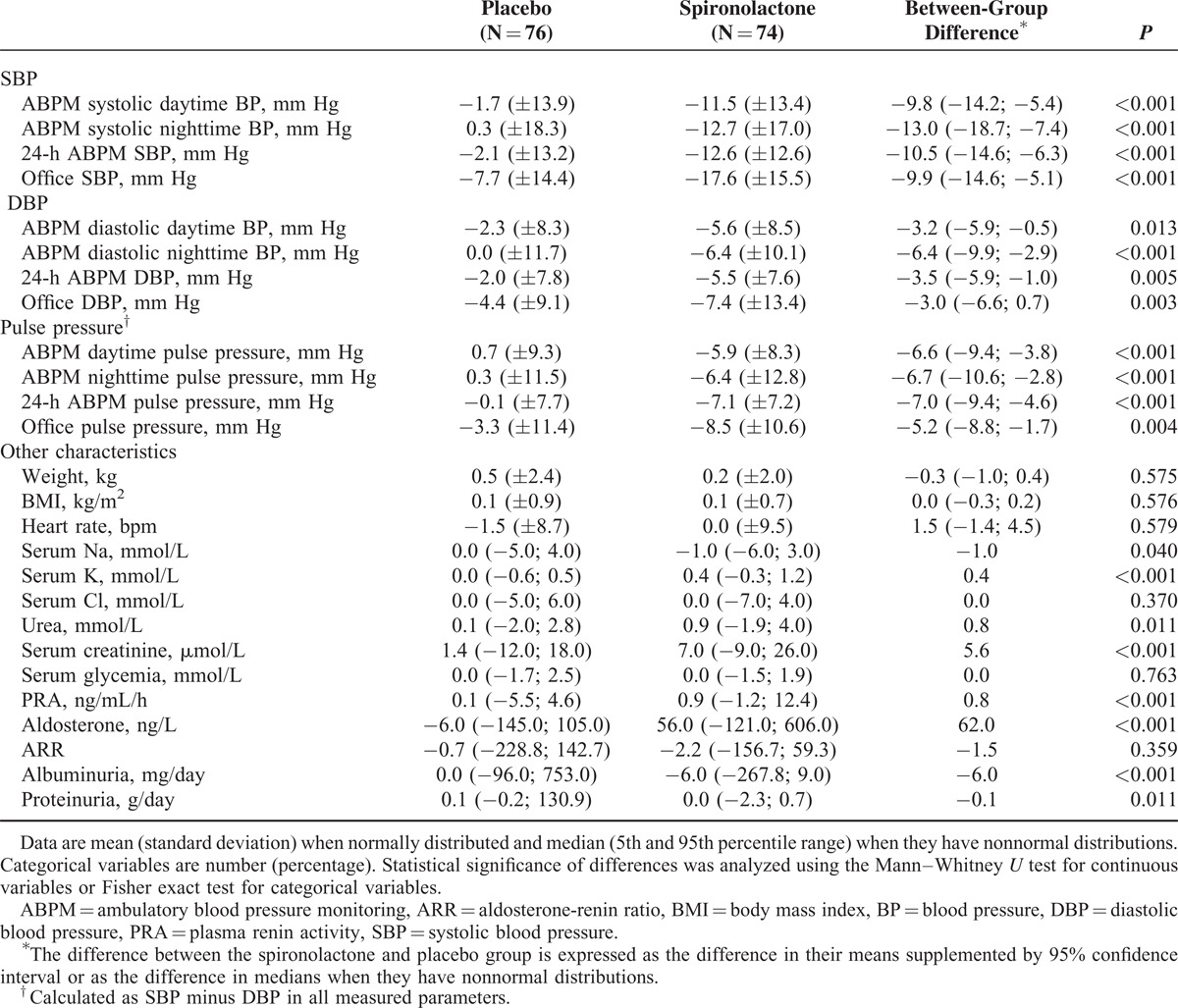
Change of Patient Characteristics at 8 Weeks Compared With Baseline

A small comparable weight gain was observed in both study groups (Table 2). With spironolactone treatment, serum sodium decreased by a median of 1.0 mmol/L, serum potassium increased by a median 0.4 mmol/L. The mean serum potassium increased during the 8 weeks of spironolactone treatment from 4.10 to 4.49 mmol/L, the highest reached serum potassium value at 8 weeks was 5.6 mmol/L. Small increases of serum urea and creatinine as well as PRA and aldosterone were observed in the spironolactone group (Table 2). No patient was excluded from the study because of severe hyperkalemia or progression of renal insufficiency. Albuminuria and proteinuria decreased with spironolactone treatment (Table 2).

The office SBP goal <140 mm Hg at 8 weeks was reached in 54 (73%) patients using spironolactone and in 31 (41%) patients using the placebo (*P* = 0.001). The respective office DBP goal <90 mm Hg was reached in 54 (73%) patients using spironolactone and 48 patients using the placebo (63%) (*P* = 0.223).

To evaluate the BP response to treatment, both the spironolactone and placebo groups were further subdivided based on the median values of potassium, aldosterone, PRA, and ARR. Both baseline potassium ≤4.1 mmol/L and baseline ARR >17 predicted larger SBP and DBP reduction with spironolactone treatment after 8 weeks (Table 3). The baseline serum aldosterone and PRA did not significantly predict BP response to spironolactone treatment (Table 3). When standard cutoffs were used, low PRA ≤1.0 ng/mL/hour predicted a good effect of spironolactone on SBP and high ARR >30 predicted a good effect on both SBP and DBP (Table 3). Response to placebo administration did not differ for any of the parameters above (data not shown).

**TABLE 3:**
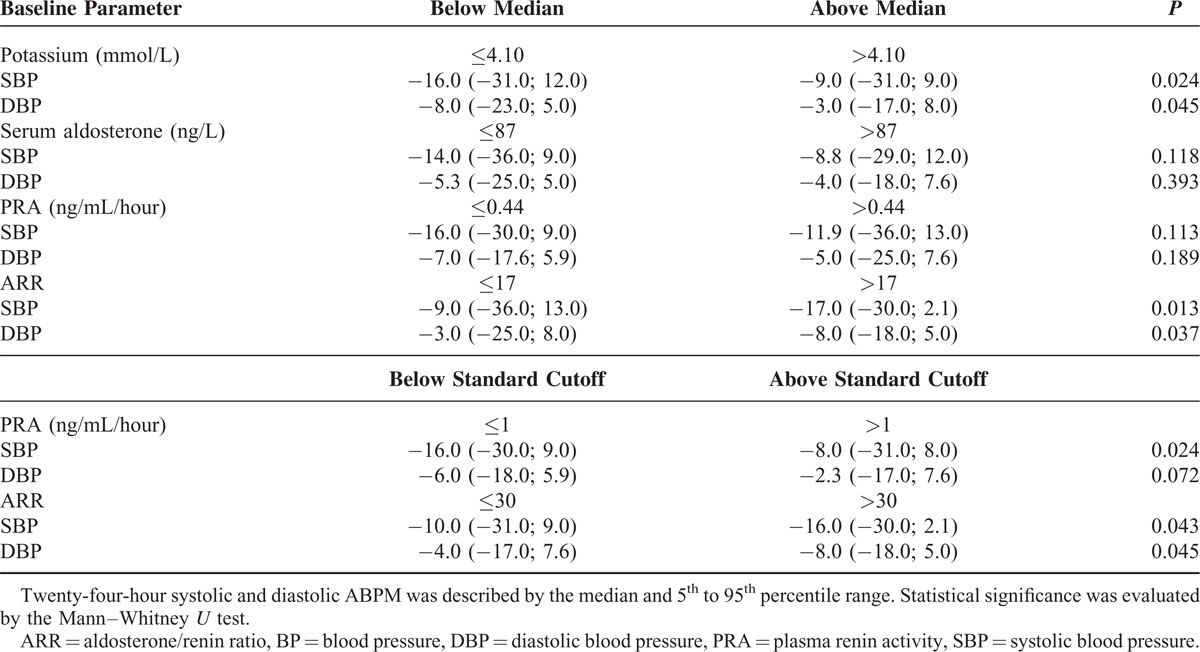
Mean Differences of 24-Hour ABPM SBP and DBP (mm Hg) After 8 Weeks of Spironolactone Treatment in Relation to Baseline Laboratory Parameters

In 40 patients (27%), a secondary cause of hypertension was found during subsequent evaluation after trial completion with comparable distribution in both study arms: primary aldosteronism (8 in the spironolactone and 12 in the placebo group), renovascular hypertension (4; 3), obstructive sleep apnea (3; 5) and nephrogenic hypertension (3; 2). The overall results of the trial were consistent when patients with secondary hypertension were removed from analyses (see eTable 1 in the Supplements, http://links.lww.com/MD/A77).

The frequency of adverse events was comparable in both study arms (see eTable 2 in the Supplements, http://links.lww.com/MD/A77). Serious adverse events leading to study medication discontinuation occurred in 4 patients using spironolactone and 1 patient using the placebo (*P* = 0.367). The total number of adverse events was 30 in the spironolactone group and 31 in the placebo group (eTable 2, http://links.lww.com/MD/A77).

## DISCUSSION

This randomized trial showed that the addition of 25 mg spironolactone daily in patients with resistant arterial hypertension using a mean of 4.5 antihypertensive drugs led to a significant decrease of SBP and DBP both in the office and during ABPM after 8 weeks of treatment. Spironolactone led to small but significant increases of serum potassium, urea and creatinine, and a decrease of serum sodium without adverse clinical consequences was well tolerated and the number of adverse effects was comparable to the placebo.

To our knowledge, this is the largest randomized placebo controlled trial to assess the antihypertensive effects of low-dose spironolactone in patients with truly drug-resistant hypertension. Previous uncontrolled observational trials showed a substantial office BP reduction after the addition of spironolactone, ranging from 14 to 32.2 mm Hg systolic and 7 to 12.5 mm Hg diastolic.^10,22^ In comparison to the previous observational trials, the magnitude of office BP fall in the spironolactone group was comparable. A fairly strong placebo effect was observed with office BP in our trial (−7.7/−4.4 mm Hg BP reduction) and, therefore, the placebo-corrected spironolactone BP lowering effect was less than reported in previous uncontrolled trials. In another randomized trial with patients with diabetes, the addition of 25 to 50 mg (average 35 mg) spironolactone led to a similar mean placebo-corrected daytime ambulatory BP decrease of 8.9/3.7 mm Hg after 16 weeks.^23^

A numerically lesser, although statistically significant, effect of spironolactone on DBP in our trial may be explained by the relatively low baseline DBP with a significant proportion of patients (37%) having isolated systolic hypertension. Besides the diuretic effect of spironolactone, its reduction of vascular stiffness probably plays an important role in patients with resistant hypertension^24^ and could explain the significant reduction of pulse pressure and larger reduction of SBP in our trial.

The strongest effect of spironolactone in the ASPIRANT–EXT trial was observed on nighttime BP (decrease of −13.0/−6.4 mm Hg), which was not affected by placebo at all. Nighttime BP is a very strong predictor of future cardiovascular events^25^ and we postulate that its marked reduction by spironolactone might provide a prognostic benefit in patients with resistant hypertension. Furthermore, the observed reduction of proteinuria and albuminuria with spironolactone is a sign of possible end-organ protection.

The dose of 25 mg spironolactone daily, chosen to be administered in our trial, seems optimal to us for use in resistant hypertension, as it offers good antihypertensive efficacy and a low number of adverse effects, comparable to placebo in the short-term. With the 25 mg dose, the long-term occurrence of adverse events is low, about 13%, leading to the discontinuation of spironolactone only in 6% of patients.^9^ There may be a dose–response effect with spironolactone up to 50 mg/day in patients with essential hypertension and higher doses >50 mg/day do not produce further reductions in BP.^26^ In patients with primary aldosteronism, increasing the dose of spironolactone (up to 75–225 mg/day) may have a greater antihypertensive effect.^27^ It is possible that the increase of spironolactone dose to 50 mg/day or more could have led to a more substantial decrease of BP, but would be likely to also cause a higher occurrence of adverse events.

A possible limitation of our study is the relatively short duration of 8 weeks. As the maximal hypotensive effect of spironolactone is reached after 7 weeks of treatment in patients with resistant hypertension,^28^ we are convinced that the 8-week study duration was sufficient for the full effect of spironolactone to be shown, and no further BP reduction would be observed if the trial had had a longer duration. However, the hormone-related adverse effects of spironolactone (such as gynecomastia) usually take a longer time to develop and would likely become more frequent with a longer study duration.^9^

Eplerenone has also been shown to provide significant add-on BP lowering effect when treating resistant hypertension. The BP reductions achieved by 50 to 100 mg eplerenone daily in an observational trial were very similar to BP reductions achieved by 25 mg spironolactone in this trial: clinic BP was reduced by eplerenone by 17.6/7.9 mm Hg and 24-hour ambulatory BP by 12.2/6.0 mm Hg.^29^ Therefore, eplerenone seems to be an appropriate alternative if spironolactone is not tolerated because of sexual adverse effects.

The mild increase of serum potassium and creatinine with spironolactone was expected. It needs to be stressed, that the majority of recruited patients had normal renal function with only 18.7% of patients exceeding the baseline creatinine upper reference limit of 104 μmol/L. The risk of hyperkalemia and worsening of renal function would be higher if spironolactone was used in patients with chronic kidney disease, especially with a glomerular filtration rate <45 mL/minute and serum potassium >4.5 mmol/L.^30^

Previous trials reported conflicting data about whether the BP response to spironolactone can be predicted by baseline aldosterone, ARR, or baseline potassium.^7,11,23,31^ In our trial, larger BP reduction was observed in patients with higher baseline ARR >17 and lower baseline potassium ≤4.1 mmol/L. This could possibly help identify the patients for which treatment with spironolactone is most effective.

Antihypertensive drugs were not discontinued before blood sampling in accordance with current guidelines,^21^ which might have affected the measured values of ARR and may be another limitation of this study, but we believe that this approach is easier to adopt in everyday practice.

Patients enrolled in our trial were obese with mean body mass index >32 kg/m^2^. Indeed, patients enrolled in a majority of recent trials in resistant hypertension were also obese or at least borderline overweight, regardless of whether the trials used spironolactone^3,6,7,9,11,23,30^ or other measures^32–34^ as treatment intervention. Obesity has recently been found to be closely connected with resistant hypertension,^4^ and in fact it appears to be a cause of treatment-resistant hypertension.^35^ We are convinced that the results of our trial can be generalized to the vast majority of resistant hypertension patients encountered in everyday clinical practice.

According to the World Health Organization, the global prevalence of hypertension is currently about 1.5 billion people and the prevalence of drug resistant hypertension is about 12%. Based on these statistics, we estimate that there are about 180 million patients with resistant hypertension worldwide. Keeping this in mind, we decided not to insist on complete exclusion of secondary hypertension before enrollment into the trial, as these measures would not be possible in certain developing countries. In our previous analyses, we showed that spironolactone treatment is effective to a similar extent both in patients with and without a secondary cause of hypertension,^36^ and its antihypertensive efficacy seemed to be higher in elderly patients age >62 years^37^. The current analysis confirmed that the presence of more than one-fourth of patients with subsequently found secondary forms of hypertension did not change the overall trial results. However, secondary causes of hypertension should be intensively looked for in patients with resistant hypertension and treated causally whenever possible.

A recent randomized sham-controlled trial (A Controlled Trial of Renal Denervation for Resistant Hypertension) did not find the catheter-based renal denervation to be more effective than the sham procedure in lowering BP in resistant hypertension.^38^ Thus, at present spironolactone appears to be the most effective measure to control BP in resistant hypertension. Whether the positive effect of spironolactone on BP leads to a decreased number of cardiovascular events and decreased mortality needs to be explored in further studies.

In conclusion, the ASPIRANT-EXT trial confirms that spironolactone is an effective drug to lower both SBP and DBP in patients with resistant arterial hypertension with excellent safety over a short period. These results warrant more widespread use of spironolactone globally, considering its low cost and wide availability.
